# Field evaluation of the impact of cocoa swollen shoot virus disease infection on yield traits of different cocoa (*Theobroma cacao* L.) clones in Ghana

**DOI:** 10.1371/journal.pone.0262461

**Published:** 2022-01-20

**Authors:** Atta Ofori, Francis K. Padi, George A. Ameyaw, Abu M. Dadzie, Michael Opoku-Agyeman, Owusu Domfeh, Frank O. Ansah

**Affiliations:** Cocoa Research Institute of Ghana, New Tafo, Akim, Ghana; Shahjalal University of Science and Technology, BANGLADESH

## Abstract

Cocoa swollen shoot virus disease (CSSVD) is a major disease of cacao (*Theobroma cacao* L.) in Ghana and other West African countries that grow the crop. Attempts to develop resistant varieties since the discovery of the disease in 1936 have yielded little success. Recently, planting materials that are tolerant to the disease have been recommended for planting in areas with high CSSVD prevalence. However, the effect of CSSVD on yield component traits of most cacao clones including the tolerant varieties has not been well studied. To investigate the impact of CSSVD on these traits, reduction in bean weight (BW), number of beans per pod (NoBP) and dry bean yield (DBY) between symptomless and symptomatic trees, and disease incidence (DI) among 210 different cacao clones were evaluated. The clones were transplanted in June 2010 following a randomised complete block design with four replications consisting of three trees per clone per block. Response of the clones to CSSVD incidence had 180 of the genotypes having < 50% symptoms. Clones of Contanama, Iquitos, Marañon, Nanay and unknown derived from Upper Amazon parentage had less DI at the end of the study. The CSSVD effect (symptomless vs. symptomatic) was significant (p < 0.05) for DBY and NoBP, and the symptomless trees surpassed on average their symptomatic trees by 21.17% for DBY. Some of the best-performing clones identified under natural CSSVD infection were; COCA 3348/52 and GU 219/V among the underrepresented clones, B 36, ICS 40, NA 33 x IMC 67, T30/628, T60/887, T63/971, T 81/1879 and T 82/503 among those that combine high DBY with low yield reduction, and NA 124, T17/358, T35/78, T57/305, T63/971 x SCA 6, T65/239, T76/1835 and T82/2294 among those that combine high DBY with no disease incidence. Their inclusion in breeding programs that seek to develop resistant CSSVD varieties or deployment as planting materials in endemic areas to improve yield production in Ghana is recommended on the basis of the present observations.

## Introduction

Cocoa production in Ghana has not matched expansion in the area of cultivated land over several decades. The lack of good cultural practices and the impact of disease and pests have frequently been cited as some of the major causes of the poor productivity in the country [1]. The cocoa swollen shoot virus disease (CSSVD) is one of the most devastating diseases on cacao, and causes significant production losses and death of cacao trees [2]. The CSSVD is caused by a badnavirus within the family *Caulimoviridae*. Several isolates/strains of the virus occurring in Ghana and other West African Countries have been characterized using molecular studies [3]. The virus is transmitted by mealybug vectors through their feeding activity as they move through the cacao canopy.

Infected cacao plants can express different symptoms in the leaves, stems, roots and pods. These symptoms include red vein-banding in young leaves which may be followed by vein clearing or chlorosis along the veins [4]. Other symptoms that may occur concurrently or after the leaf symptoms are swellings in stems, roots, and chupons, and also distortion in pod shape and size, with affected pods becoming almost round or spherical [5]. All these symptoms negatively influence the physiological response of the cocoa tree.

Cocoa Swollen shoot disease control in Ghana starts with identification of trees infected with the disease by trained disease spotters who rely on the various visual symptoms. In a farm with CSSVD outbreak, the rate of infection and amounts of infection relate to the combined effects of resistance of genotype to infection and length of the incubation period between infection and virus availability for transmission [6]. The time of infection and expression of symptoms may vary from genotype to genotype, depending on latency period. In some cacao genotypes, it may take two years before an infected tree will show symptoms [7].

The only effective treatment for CSSVD is eradication, where the infected plant or farm are cut and replanted with disease free planting materials. This method is however very expensive and currently a little over 300 million diseased cacao trees have been cut since the launch of the program in 1946 [8]. Besides spending huge amount of money in cutting the trees, the Government of Ghana is deprived of revenue which could have accrued from cacao yields coming from these farms. The farmers who are also directly involved lose their regular source of income [9].

In addition to the eradication method, several interventions such as biological control of the CSSV mealybug vector [10], removal of alternative host plants [11], and barrier cropping through isolation of cacao farms [12–14] have been promoted across time to reduce the spread of the disease. Studies [11, 14] however show that the expected reduction in the spread of the CSSV disease has not been achieved and the disease continues to spread at an alarming rate. Inadequate funding for the interventions and low adoption rate of measures recommended for CSSVD control by farmers are some of the reasons cited for the limited success of the programs [8]. With these interventions proving ineffective to control the disease, alternative measures such as resistance breeding, which has long been considered as the most sustainable long-term solution to the disease, is currently being advocated [15, 16].

The first CSSVD resistance study conducted were on Amelonado varieties that formed the basis of successful West African cocoa industry and was found to be highly susceptible to the CSSV disease [17]. This led to the introduction of the more vigorous Upper Amazon germplasm, which even though were not resistant were found to possess higher tolerance to CSSVD than Amelonado when progenies of Amelonado and the Upper Amazon populations were tested [18, 19]. The cacao progenies currently cultivated in most of the cacao-growing regions in West Africa are predominantly of Upper Amazon origin [20, 21].

The yield of cacao in Ghana and West Africa, at large, has generally been considered very low even though most farms have been replanted with Upper Amazon varieties which are tolerant to CSSVD disease. In Ghana, annual average yields are typically in the range of 400 to 450 kg/ha [22], though on-station trials and on-farm demonstrations have recorded dry bean yields in excess of 2,000 kg/ha [23]. Although most farms are now planted with the Upper Amazon varieties, recent surveys show that the Upper Amazon varieties succumb to the CSSVD under plantation conditions [13, 14, 24]. The inconsistency in performance of the Upper Amazon planting materials may be partly accounted by the methods used in selecting those materials. Most of the studies that selected CSSVD resistant materials were carried out on young cacao seedlings under gauze-house conditions using vector transfer techniques or seed inoculation method [25]. These methods do not portray natural field conditions as yield, which is the most important component of the crop, is not considered. Selecting genotypes that are high yielding under natural CSSVD conditions is important for sustainable cacao productivity.

Despite the economic importance of CSSVD in West Africa, very few of the cacao clones have been evaluated for their tolerance to CSSVD. The cacao species has traditionally been grouped into Trinitario, Criollo and Forastero, based on morpho-geographic classification [26], until about a decade ago when [27] proposed ten genetic groups, namely, Amelonado, Contanama, Criollo, Curaray, Guiana, Iquitos, Marañon, Nacional, Nanay, and Purús. Across time, highly diverse accessions have been collected in the primary center of diversity and distributed to various cacao gene bank centers, such as the Centro Agronomico Tropical de Universal Investigacion Ensenanza (CATIE) in Costa Rica, the International Cocoa Gene bank (ICG) in Trinidad, and the International Cocoa Quarantine Centre, UK. In Ghana, different cacao introductions from these gene banks have occurred over different time scales and currently, 959 germplasm accessions are being conserved in a field gene bank at the Cocoa Research Institute of Ghana (CRIG).

In Ghana and most of West Africa countries where the yield response of most cacao clones to CSSVD is unknown, it is important to comprehensively investigate the effect of CSSVD on yield performance of cacao clones. The yield of cacao is a complex trait and depends on components such as bean weight, number of beans per pod and number of pods per tree, which could also be influenced by numerous environmental factors such as pollinations and diseases [28]. This paper therefore emphasizes the importance of estimating the effect of CSSVD on yield component traits in cacao. It demonstrates that in addition to the requirement of gauze house screening where seedlings are infected with CSSV, the final selection of CSSVD tolerant material must be based on responses of yield component traits to CSSVD infection. The relative reduction in yield component traits of symptomless and symptomatic trees and disease spread among 210 different cacao clones were evaluated. Broadening our knowledge on how CSSVD infection influences yield component traits is necessary for the cacao industry in Ghana. This will guide the selection of high yielding and good bean quality cacao clones that are tolerant to CSSVD in Ghana.

## Materials and methods

### Plant materials

A total of 210 cacao clones were selected for this study. These comprised clones from different genetic origin (Table 1) and selected on the basis of differences in their introduction period and breeding history. This includes clones introduced before the beginning of formal research in 1938 which were mostly the local Amelonado, the most susceptible genotype and the local Trinitario introduced by Tetteh Quarshie and Governor Griffiths, respectively [29]. The Trinidad introductions clones (referred to as T) which comprises different combinations of open and hand pollinated pods from the upper Amazon, lower Amazon and Trinitario genetic group, and brought into Ghana by Posnette in 1944 [29]. The British introductions which consisted of true to type clones of Nanay (Na), Iquitos (IMC), Maranon (PA), and Contanama (SCA) cover introductions made between 1960s to 1980s [30]. The relatively recent introductions largely comprised the Guyana (GU), Nacional, Curaray and Purus genetic group [31]. Clones that could not be allocated to any of the genetic groups including hybrids were classified as unknown. Five seed garden clones (T60/887, T63/971, T17/358, T85/799 and T79/501) that are tolerant to CSSVD and high yielding [32] were included.

**Table 1:** Genetic type of 210 cocoa clones used for the study.

Genetic type	Clone
Amelonado	A 196, BE 10, CC 10, CC 11, DOM 4, MA 12, P 30, TF 6, TF 20
Nacional	MO 9, MO 20, T56/118 (MO 14 open-pollinated)
Iquitos	IMC 6, IMC 23, IMC 45, IMC 53, IMC 55, MAN 15/2, T16/530 and T16/618 (IMC 24 open pollinated), T17/358, T17/1856 (IMC 53 open pollinated), IMC 60, IMC 61, IMC 67, IMC 76 IMC 83, AMEZ 3/2
Curaray	COCA3348/52, LCTEEN 261/54
Guiana	GU 123/C, GU147/C, GU 125/V, GU 219/H and GU249/H
Nanay	NA 3, NA 33, T11/94 (NA 33 open-pollinated), T14/233, T14/295 (NA 43 open-pollinated), T13/472, T 13/382 and T 13/383 (NA 60 open-pollinated), NA 124, NA 227, NA 427, NA 242, NA 387, NA 535, NA 904, NA 929, Pound 7, Pound 12/A
Purus	RB 41, RB 49
Marañon	PA7, PA 13, PA 16, PA 37, PA 56, T53/46 (PA 37 open-pollinated), PA 65, PA 70, T30/539 and T30/628 (PA 103 open-pollinated), PA 107, PA 118, PA 124, PA150
Unknown	EET 397 and EET 399 (Ecuador), RIM 189 (Mexico), SGU 50 (Criollo x Matina hybrids), T60/887, T60/885, T60/1052, T60/1774 and T60/975 (PA 7 x NA 33), T60/887 x IMC 53, T60/887 x NA 33, T60/887 x PA 7/808, T61/1239 and T61/13/26 (NA 33 x NA 32), T62/205 (NA 33 x NA 34), T63/762, T63/961, T63/971, T63/967, T63/852 and T63/882 (PA 35 x NA32), T63/971 x SCA 6, T65/236, T65/238 and T65/329 (PA 7 x IMC 47), T72/2388 and T72/1768 (NA 3 x IMC 60), T73/1931 and T73/2454 (NA 33 x IMC 60), T76/1068, T76/1136, T76/1224 and T76/1835 (PA 35 x NA 31), T79/1150, T79/380, T79/1064 and T79/467* (NA 32 x PA 7), T81/1879 and T81/1880 (NA 31 x NA 32), T82/503 and T82/2294 (NA 32 x PA 35), T85/874 and T85/799 (IMC 60 x NA 34), T85/799 x MA 12, T87/98, T87/12 and T87/368 (IMC 60 x NA 34), T88/2130 (ICS 85 self), T90/114, T90/187 and T90/2093 (IMC 76 x NA 32), T92/795 and T92/1614 (NA 32 x NA 31), T99/129 and T99/395 (ICS 56 x ICS 85), NA 33 x IMC 53, NA 33 x IMC 67, NA 33 X PA 7, NA 33 x T60/887, PA 150 x IMC 67, PA 150 x NA 33, PA 150 x SCA 6, PA 150 x SCA 9, PA 150 x T60/887, PA 7 x IMC 53, PA 7 x IMC 67, PA 7 X NA 33, PA 7 x T 60/887, C/SUL 7, EQX 78, EQX 3338, GC 29, MOQ6/95, MOQ 210, PASCAL, PNG 10, PNG 360, PNG 336, T 4/159, T 6/526, T 6/525, T 8/287, T 8/199, T 9/66, T 114/584, T 20/126, T 20/50, T 23/458, T 23/509, T 28/805, T 29/378, T 35/78, T43/1054, T44/547, T89/158
Contanama	SCA 6, SCA 9, T12/61, T12/63, and T12/151 (SCA 12 open-pollinated)
Trinitario	A 12, A 46, A 45, A 72, A 164/43, A 164/45, B 36 (local trinitario), CAM 12 (trinitario), D 26, D 70, T3/247 and T3/335 (local trinitario) ICS 16, ICS 25, ICS 39, ICS 40, ICS 43, ICS 70, E 9, E 9/195, E 17, E 75, K 5, K 9, O 2, R 15 (local trinitario), S 19, S 72, T24/229 and T24/297 (ICS 80 open-pollinated), T26/277 (ICS 45 open-pollinated), T39/659 (ICS 6 open-pollinated), T45/145 (ICS 70 open-pollinated), T57/305, T57/308 and T57/368 (ICS 60 open-pollinated), ICS 70, T88/2130 (ICS 85 SELF) U 7, Y 44 and Z 47 (local trinitario)

*Unknown—Clones that could not be allocated to any of the genetic groups which includes hybrids.

### Field evaluation and plant culture

Field experiment was conducted at the Cocoa Research Institute of Ghana (CRIG), Tafo (a humid rainforest belt, with latitude 06^0^ 13΄N, 0^0^ 22΄ W). Soil samples randomly taken from the experimental site and analysed before planting indicated 0.9 g/kg nitrogen, 8.6 g/kg carbon, 7.08 (μg/g) available phosphorus and 0.60 (meq/100g) potassium. In generating experimental materials, scions from fan branches of the 210 selected clones were budded onto six-month old rootstocks generated from PA7/808 x PA 150. The developed clones were transplanted in July 2010, eight months after budding, following a randomized complete block procedure with four replications, consisting of three trees per clone per block. They were planted at 3.0 m^2^ spacing under both plantain as temporary shade and *Terminalia spp*. as permanent shade. The plantain was planted at 3.0 m^2^ spacing and the permanent shade at 18.0 m^2^ spacing. The shade plants were planted three-months before the cocoa clones were transplanted. Standard crop management practices for weed, pests and disease control, and fertilizer application were followed.

A visit by CSSV disease inspection team in June, 2013 revealed that some of the trees showed symptoms of CSSVD even though there was no cacao farm around the area and no deliberate CSSV inoculation was made. A thorough field monitoring revealed that the presence of CSSVD symptoms was clone specific ie GU 225V, R 15A, RB 45, ALPHA B36, ICS 95, 10P, CAS 3, PA 140, T16/613, T85/787 and T44/546. Tracing the source of budwood of these clones indicated that the parent trees had CSSVD infection. Natural spread of the disease from trees of these clones to others within the plot was subsequently monitored.

### Field data collection

The spread of infections from the infected clones to other trees was monitored by a disease spotter with years of experience in CSSVD identification. Visual symptoms such as red vein banding, vein clearing or chlorosis along the veins, swelling in stems and chupons were assessed. Even though CSSVD was identified in the plot in June, 2013, data collection was delayed until January, 2015 to allow canopy formation to facilitate transmission of virus by mealybugs. Monitoring of CSSVD symptoms of the whole plot was on a bi-monthly basis until December 2020, when about 30% of the test trees had symptoms and infected trees were visibly labeled and recorded as follows;

- Date of first appearance of visible symptoms of CSSVD in a tree
- Type of symptom expressed in a tree

Percentage disease incidence per clone was estimated by dividing the total number of trees of a clone showing symptoms with the total number of trees of that same clone and multiplying by 100 at the end of the evaluation period for all the 210 clones. When estimating the yearly proportion of disease incidence, the available trees were taken as symptomless until a tree expressed symptoms. The percentage disease incidence was further classified as no-disease incidence (0%), low disease incidence (<25%), moderate disease incidence (25–50%), high disease incidence (50–75%) and very high disease incidence (>75%) for the different cocoa genetic groups. Apparent rate of virus spread in the field was estimated by regressing yearly proportions of disease incidence on time.

Yield data collection was carried out between August 2018 and April 2020. In order to avoid biased results, clones were checked thoroughly and those with 20% tree death or having almost all trees showing symptoms were excluded. Two clones, CAM 12 and PA 124 among the 29 no-disease incidence class were also excluded because they could not produce enough pods for yield component analysis. Sixty eight clones, 27 from the no-disease incidence class and 41 from the moderate disease incidence class (ie clones with substantial proportions of symptomless and symptomatic trees for comparison) were evaluated. At harvest, the number of mature pods of clones from symptomless and symptomatic trees was bulked separately, counted and broken for each plot. Dry mass of seeds, obtained from a sample of 20 pods per group per clone after being fermented and dried to a moisture content of 7%, was used to estimate the mean bean weight after counting. Pod value (number of pods needed to produce 1 kg of dry beans) was estimated from the mean bean weight and number of beans per pod. Total yield was determined from the total number of pods produced per clone per group divided by their pod values.

### Indexing of symptomless trees

To determine if some of the seemingly healthy trees could also have been infected, some of the symptomless cacao trees were indexed after the sixth year when 30% of the trees were showing symptoms. Three budwoods were taken from selected tagged trees and patch-budded onto Amelonado (the most susceptible cacao cultivar) seedlings and subsequently observed for symptoms of CSSVD. The standard procedure for CSSVD indexing adopted by [13], where symptoms are visibly seen in tender new leaves of infected trees within nine months after budding was followed. Percentage latency, the apparently healthy trees in the field that showed symptoms after indexing and reliability of visual identification, the apparently healthy trees in the field that did not show symptoms after indexing were estimated.

### Statistical analysis

Analyses of variance (ANOVA) were first run separately for percentage disease incidence of the 210 clones, yield of the 27 clones from the no-disease incidence class and 41 of the moderate and high disease incidence class using GenStat statistical software version 11(VSN International Ltd., Hemel Hempstead, UK). The average trait values, with clones considered fixed was computed for each trait using a random effect model. Normality of each data was checked based on the plot of the residuals. For the 41 clones, sum of squares for entry was partitioned into symptomless vs. symptomatic effects. In estimating relative reduction of yield traits among clones, mean differences (MD) in percentages between symptomless and symptomatic trees of same clones were calculated as follows:

MD=(SL−S)×100/SL,

Where; SL is symptomless and S is symptomatic. Treatment means were separated via the Duncan’s multiple range test at *p* = 0.05 level of significance.

## Results

Classification of the 210 clones based on percentage disease incidence revealed that 29 of them did not show any symptoms during the study period (Table 2, S1 Table). Among the 29 clones, Nanay clones had two, Marañon had two, Iquitos had three, Contanama had one, Trinitario had five and Unknown clones had 16. Of those clones that showed symptoms, clones of Nanay, Marañon, Iquitos, Contanama Guiana, Curaray, Nacional and Purus origin generally dominated the low DI class < 50%. Trinitario and the Unknown clones had substantial numbers in all the classes even though those with lower DI, < 50% were more than those with higher DI, > 50%. The Amelonado clone dominated the high CSSVD incidence classes. In general, the distribution of DI had 180 out of the 210 clones having infections lower than 50% (Table 2).

**Table 2:** Number of clones per genetic group with disease incidence in a given range and percentage symptomless clones evaluated in field.

Genetic group	Number of clone	Number of clone per genetic group showing CSSVD incidence					% symptomless clone
		0%	Low (<25%)	Moderate (25–50%)	High (50–75%)	Very high (>75%)	
Amelonado	9	0	1	4	2	2	0
Guiana	5	0	2	3	0	0	0
Curaray	2	0	1	1	0	0	0
Iquitos	16	3	5	5	2	1	16.6
Nanay	18	2	5	8	2	1	10.5
Marañon	14	2	5	6	1	0	14.3
Contanama	5	1	2	2	0	0	20
Trinitario	41	5	12	13	7	3	12.1
Purus	2	0	1	1	0	0	0
Nacional	3	0	1	3	0	0	0
Unknown	95	16	29	32	12	6	16.
Total	210	29	64	78	26	13	

Unknown- clones which cannot be assigned to any of the identified genetic groups.

**Fig 1:**
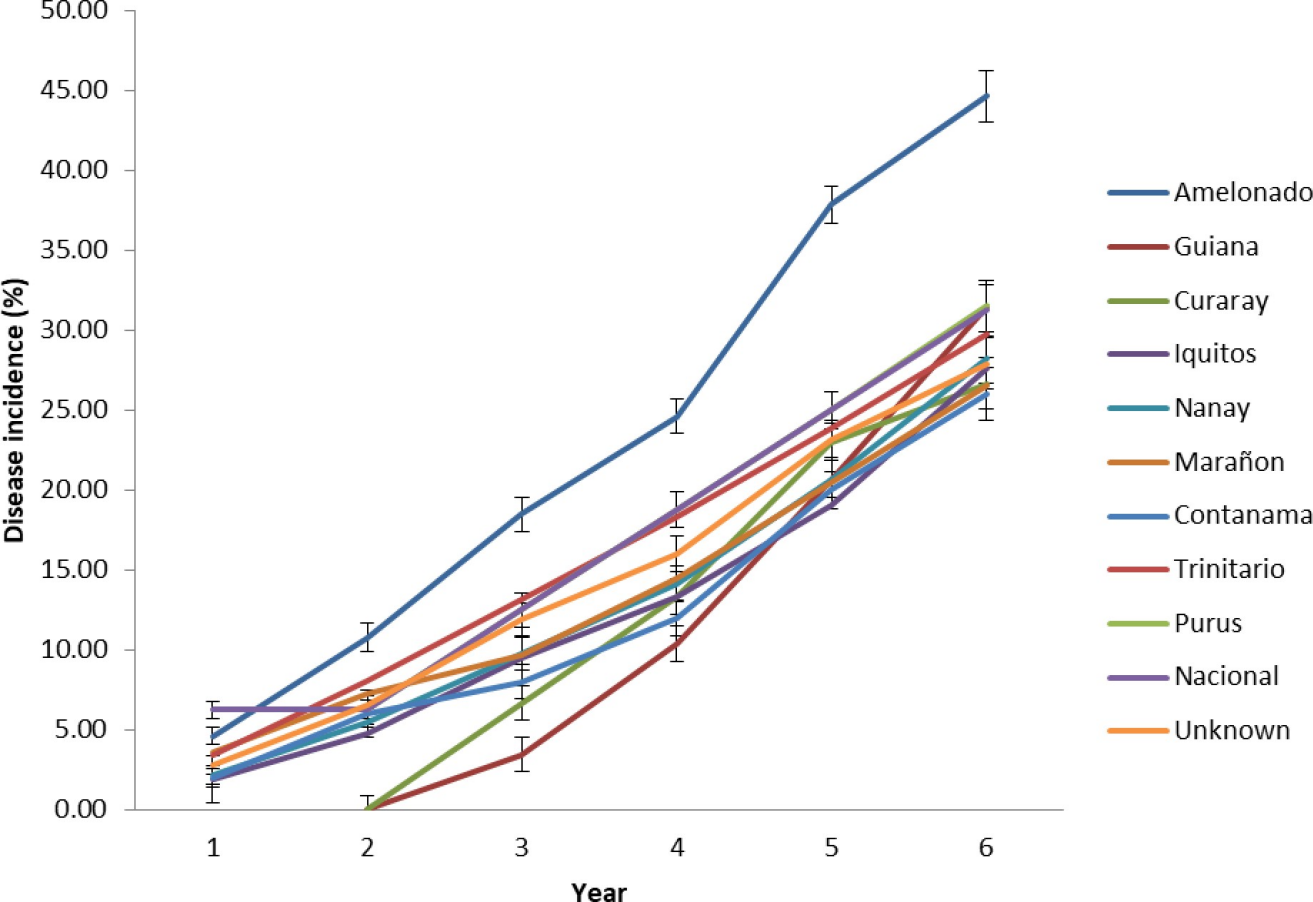
Disease incidence (%) over time among the different genetic groups over six—year period. Bars indicate standard error of means.

Significant differences were found among the different cacao genetic groups (Fig 1) in symptom expression. The first year DI was significantly (p < 0.05) higher in Nacional (6.3%) clones and lowest in the Iquitos (1.90%) clones. Disease incidence in Guiana and Curaray clones were delayed until the second year. Though DI progressed in all the groups over time, increments were high in Amelonado. Clones of Contanama had the lowest (26%) DI and the highest was Amelonado (44.8%) by the end of six-year data collection period.

There was a very high level of variation among the 27 clones of the no-disease incidence class evaluated for dry bean yield (DBY) with more than three-fold difference between the least-yielding clone (T9/22–0.72 t/ha) and the highest-yielding clone (T63/97 x SCA 6–2.1 t/ha) (Table 3). Six of the clones (T76/1835, NA 124, T82/2294, T65/239, T35/78 and T63/97 x SCA 6) were superior in yielding ability relative to the best CSSVD tolerant Seed Garden clone (T17/358–1.44 t/ha) included in the study.

**Table 3:** Mean yield of 27 clones from the no-disease incidence class evaluated over two-year period.

Selection	Yield (t/ha/year)
T 9/22	0.72
IMC 61	0.79
T 65/326	0.88
T 92/1614	0.89
T 16/618	0.90
T 20/126	0.91
T 60 x IMC 53	0.93
PA 150 x SCA 6	1.01
T 24/297	1.05
E 9	1.06
PA 118	1.10
T12/151	1.14
NA 33	1.23
T20/50	1.27
T65/238	1.27
T85/874	1.28
T49/778	1.31
T79/1150	1.34
O2	1.38
T57/305	1.40
T17/358	1.44
T76/1835	1.52
NA 124	1.64
T82/2294	1.72
T65/239	1.86
T35/78	1.92
T63/97 x SCA 6	2.06
LSD (5%)	0.78

LSD = Least significant difference.

Among the 41 clones evaluated for effect of CSSVD on yield component traits, significant differences were observed for all the traits (Table 4). Mean distribution of bean weight (BW) was 1.16 g, ranging from 0.78 g in clone SCA 6 to 1.45 g in clone ICS 25 among symptomless trees. For the symptomatic trees, the mean BW was 1.13 g and varied from 0.72 g in clone SCA 6 to 1.36 g in clone D 26. Twelve out of the 41 clones had significantly (p < 0.05) higher BW in the symptomless trees than the symptomatic trees. Clone T23/509 had the greatest influence under CSSVD infection showing both a low BW of 0.84 g and, subsequently, the highest percentage reduction (20.0%) for BW.

**Table 4:** Mean performance between symptomless and symptomatic trees for bean weight, number of beans per pod, bean yield and relative reduction in yield of 41 cacao clones evaluated over two-year period.

Clone	Average bean weight (g)			Average number of beans per pod			Average bean yield (t/ha/year)			% yield reduction
	symptomless	symptomatic	DF	symptomless	symptomatic	DF	symptomless	symptomatic	DF	
A 196	1.08	1.06	0.02	32.76	28.86	3.90**	1.18	1.03	0.16	13.10
B 36	1.08	0.95	0.13**	24.54	25.10	-0.56	1.55	1.16	0.39	25.06
D 26	1.36	1.36	0.00	28.15	29.52	-1.37	1.38	1.03	0.35	25.05
D 70	1.12	1.16	-0.04	33.89	30.50	3.39**	1.19	0.67	0.52	43.18
ICS 25	1.45	1.34	0.11*	30.70	24.53	6.17**	1.54	1.00	0.54*	34.96
ICS 40	1.31	1.25	0.06*	37.75	30.43	7.32**	2.60	2.12	0.48	18.45
IMC 76	1.13	1.12	0.01	34.57	35.16	-0.59	1.27	1.06	0.21	16.61
MAN 15–2	1.09	1.12	-0.04	32.70	30.38	2.32*	0.89	0.90	-0.01	-1.13
L 6/428	1.09	1.00	0.09*	27.08	29.75	-2.67	0.58	0.59	-0.01	-0.17
N8/112	1.01	0.97	0.04	31.91	31.58	0.33	0.81	0.49	0.31	38.67
NA 227	1.04	0.98	-0.06	34.09	27.10	6.99**	1.13	0.36	0.77*	68.23
NA 33 x IMC 67	1.10	1.16	-0.10	34.61	36.92	-2.31	1.54	1.66	-0.12	-7.86
NA 929	1.05	1.07	-0.03	30.41	28.38	2.03*	0.77	0.92	-0.14	-17.76
P 30	1.32	1.09	0.23**	27.96	27.25	0.71	0.96	0.71	0.25	25.99
PA 150 X SCA 9	1.18	1.19	-0.01	32.54	30.04	2.50*	1.65	1.10	0.54*	32.99
PA 65	1.12	0.97	0.15**	27.96	24.30	3.66**	0.66	0.79	-0.13	-19.85
PA 7	1.15	1.04	0.10*	28.99	27.85	1.14	1.25	1.09	0.16	12.79
PA 7 x IMC 53	1.19	1.23	-0.04	37.14	40.92	-2.15*	1.07	0.87	0.19	17.99
PA 7 x IMC 67	1.28	1.30	-0.03	30.66	30.33	0.33	1.14	0.82	0.32	28.52
PA 7 x NA 33	1.21	1.17	0.04	22.56	19.89	2.67*	1.43	1.14	0.28	20.13
PA 70	1.23	1.20	0.03	23.79	20.86	2.93*	0.74	0.69	0.05	6.51
PASCAL	1.18	1.13	0.05	33.86	27.59	6.27**	1.34	1.32	0.03	1.79
PENTAGONA	1.05	1.07	-0.03	25.58	23.29	2.29*	0.37	0.35	0.02	4.59
RB 41	1.13	1.14	-0.01	30.60	28.49	2.11*	1.18	1.07	0.11	9.61
RB 49	0.91	0.99	0.07*	36.94	35.63	1.31	0.59	0.65	-0.09	-15.02
SCA 6	0.78	0.72	0.06	26.90	25.54	1.36	0.46	0.25	0.21	46.32
SGU 50	1.09	1.11	-0.02	26.46	29.17	-2.71*	0.67	0.99	-0.33	-29.73
T 23/509	1.05	0.84	0.21**	44.57	39.29	3.65*	0.76	0.49	0.27	35.34
T 29/378	1.26	1.27	-0.08	37.95	33.54	4.41**	1.66	0.88	0.77*	46.75
T 3/247	1.10	1.20	-0.10	36.16	33.82	2.34*	1.61	1.04	0.57*	35.39
T 30/628	1.20	1.25	-0.05	26.98	26.70	0.28	1.49	1.29	0.19	13.32
T 39/651	1.10	1.16	-0.06	28.24	28.26	-0.02	1.96	0.93	1.04*	52.88
T 60/887	1.25	1.21	0.04	35.00	34.50	0.50	2.02	1.86	0.16	7.98
T 63/882	1.42	1.15	0.27**	36.23	37.21	-0.98	1.69	1.18	0.51	30.01
T 63/971	1.10	1.12	-0.02	30.00	31.00	-1.00	1.50	1.63	-0.13	-8.59
T 76/1136	1.35	1.25	0.10*	36.18	37.67	-1.49	2.12	1.58	0.54*	25.39
T 76/1224	1.08	1.11	-0.03	36.66	30.15	6.51**	1.47	0.83	0.64*	43.49
T 79/380	1.14	1.12	0.02	26.96	25.10	1.86*	1.03	1.19	-0.16	-15.54
T 81/1879	1.11	1.13	-0.02	38.74	32.76	5.98**	1.75	1.53	0.22	12.94
T 82/503	1.26	1.27	0.00	35.99	34.28	1.71*	2.24	1.77	0.46	20.69
TF 6	1.16	1.06	0.09*	35.03	30.98	4.05**	1.59	0.56	1.03**	64.63
Mean	1.16	1.13	0.03	31.95	30.11	1.73	1.29	1.01	0.28	21.17
LSD (5%)		0.07		1.52			0.19			

LSD = Least significant difference, DF = difference between symptomless and symptom within clones.

With regards to number of beans per pod (NoBP), symptomless trees had a mean of 32 and varied from 22.6 in clone PA 7 x NA 33 to 44.6 in clone T 23/509, whereas symptomatic trees had a mean of 30, and ranged from 20 in clone PA 7 x NA 33 to 41 in clone PA 7 x IMC 53. Out of the 41 clones, 24 were significant (p < 0.05), with 22 being higher in the symptomless trees and only two in the symptomatic trees for NoBP, indicating that the trait may be influenced by CSSVD. The mean percentage reduction across clones was 5.5% and the clone with the highest reduction (21%) was NA 229 for NoBP (Table 4).

Dry bean yield was least in Pentagona (0.37 t/ ha) and highest in ICS 40 (2.6 t/ ha) among the symptomless trees, and least in SCA 6 (0.28 t/ ha) and highest in ICS 40 (2.12 t/ ha) among symptomatic trees. Out of the 41 clones, 11 had significantly (p < 0.05) higher DBY in symptomless trees compared to the symptomatic trees. The mean percentage reduction across clones was 21.17%, and clone NA 227 that had the highest reduction in NoBP also had the highest of 68% for DBY (Table 4).

**Fig 2:**
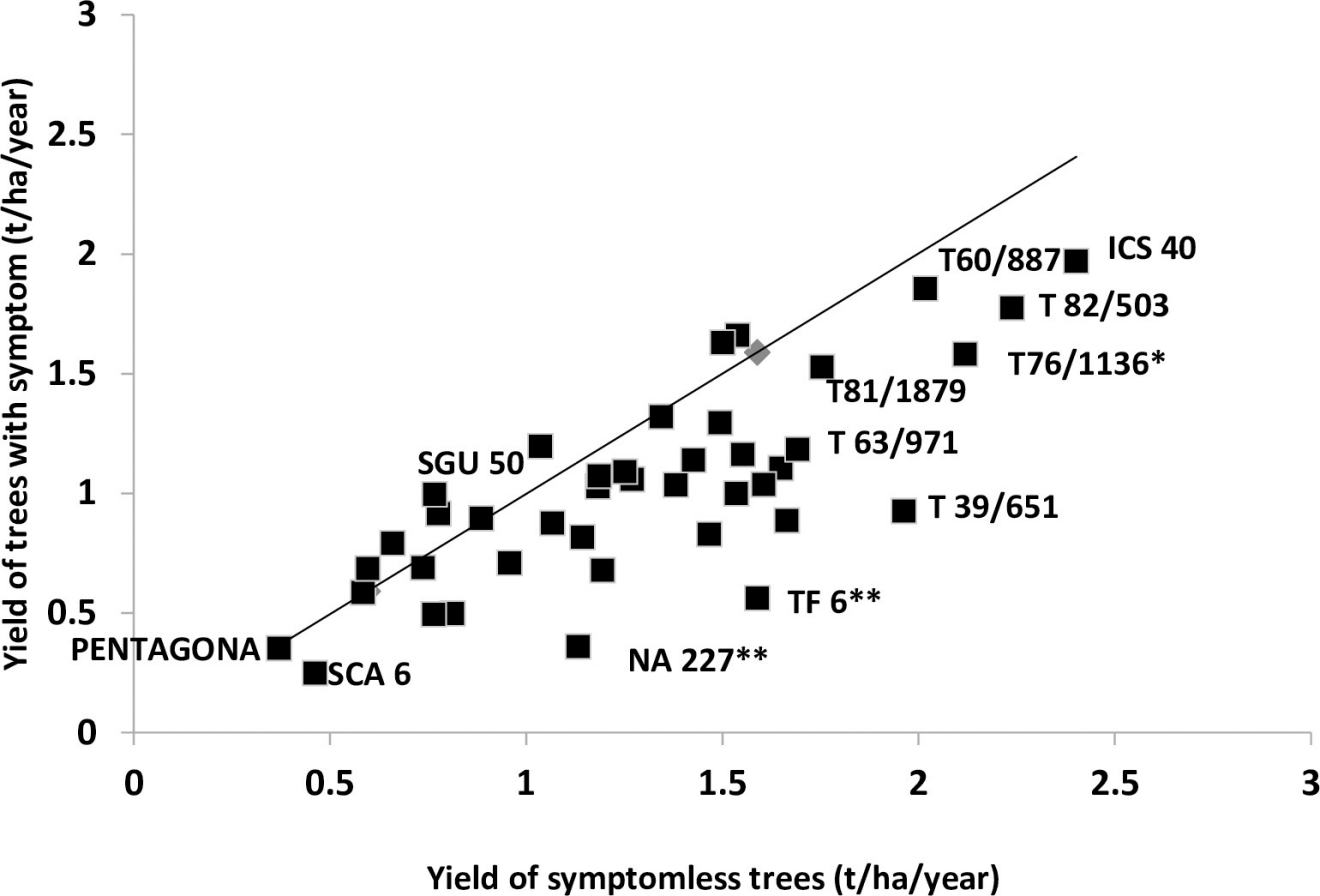
Relationship between yield of symptomless and symptomatic trees of 41 clones evaluated over two-year period. Note, deviations from the 1: 1 line due in part to yield reduction effects (R^2^ = 0·62).

**Fig 3:**
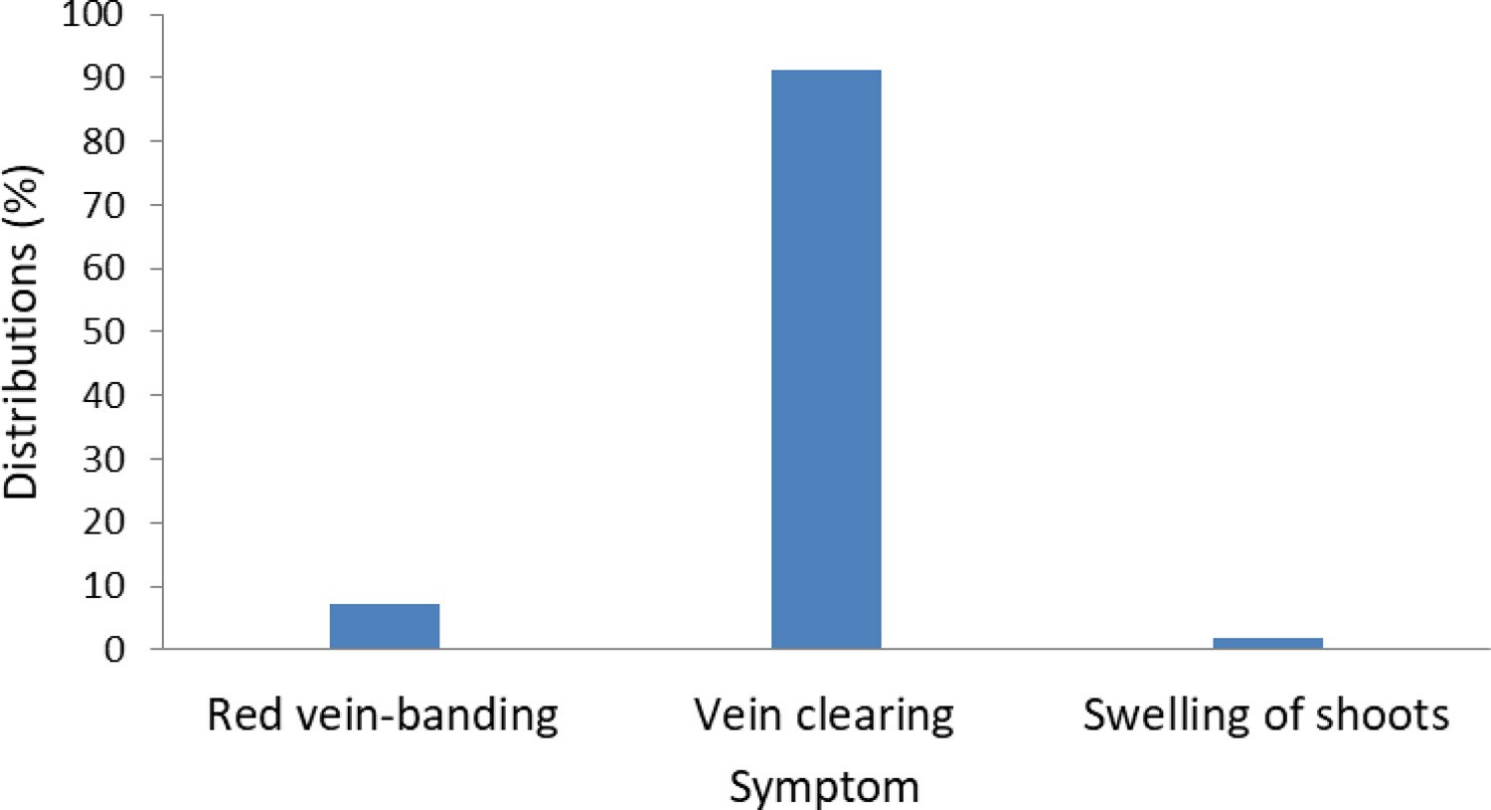
Percentage distribution of CSSVD symptoms observed during the evaluation period.

Regression of symptomatic trees on symptomless trees for DBY (Fig 2) was only moderately efficient, with coefficient of determination of 62%. Large deviations from the 1:1 line are indicative of the positive or negative response of DBY to disease infection. Of the 41 clones studied, 32 were negative indicating reduction in DBY and 9 were positive, showing increase in DBY. One of the two clones (SGU 50) with higher NoBP was among the 9 clones with increased DBY after infection. Among the four highest yielding clones (T60/887, ICS 40, T76/1136 and T82/503), T76/1136 had significant reduction in DBY between symptomless and symptomatic trees. The symptoms observed during disease monitoring period were red vein banding, vein clearing and swelling of shoots (Fig 3). Majority of the symptoms were however vein clearing (91%) even though all the symptoms were of the new Juabeng 1A strain virus which has infected cacao farms in the Eastern Region of Ghana.

Following nine months of indexing of the 29 symptomless clones, five (E 9, T 24/297, T 49/778 -Trinitarios, T 17/358 an Iquitos, T 65/238 a Nanay and Iquitos hybrid) showed symptoms (Table 5). This brings to 24 the number of symptomless clones in the study. The trees successfully selected as symptomless, which can be assumed as reliability of the visual method of identification was 92%.

**Table 5:** Indexing of 29 symptomless cacao clones in gauze house for 9 month—period.

Clones	Total number of trees grafted	Number of grafted trees showing symptoms (9M)	Clones	Total number of trees grafted	Number of grafted trees showing symptoms (9M)
CAM 12	3	0	T 9/22	3	0
E 9	2	2	T 35/78	2	0
IMC 61	2	0	T 49/778	3	1
NA 124	3	0	T 57/305	2	0
NA 33	3	0	T 60/887 × IMC 53	4	0
O2	4	0	T 63/971 × SCA 6	3	0
PA 118	5	0	T 65/238	4	1
PA214	4	0	T 65/239	3	0
PA 150 × SCA 6	4	0	T 65/326	3	0
T 12/151	4	0	T 76/1835	4	0
T 16/618	4	2	T 79/1150	2	0
T 17/524	3	0	T 82/2294	2	0
T 20/126	2	0	T 85/874	2	0
T 20/50	3	0	T 99/1614	3	0
T 24/297	2	1			
Total				88	7
Reliability of visual identification (%) = 92.0					

## Discussion

CSSVD is a major threat to cacao production in West African countries and, the expectation in Ghana is to progressively remove about 100,000 ha of infected CSSVD outbreaks across the cocoa regions by 2023 [32]. Efforts to re-establish most of the infected cacao farms with cacao varieties that are tolerant to the disease have increased currently in Ghana. However, information on the effect of CSSVD infection on yield components of most cocoa clones is limited even though they are important quality traits for selection of tolerant cacao varieties. The focus of this study was to provide information on the effects of CSSVD on yield component traits and the rate of spread of the disease among different cacao genetic groups.

It is noteworthy that the current study was established in 2010, but data on yield component traits were collected in 2018/19 and 2019/20 cropping seasons. This is because to estimate tolerance, substantial number of trees from symptomless and symptomatic trees of the same clone is required for comparison and it was at that stage that about 30% of the clones had symptoms. The yield data collection was also done for two years because some of the symptomless trees selected later showed symptoms. This reduced the number of trees in the symptomless group, making data collection difficult after the 2019/20 cropping season. Results from the study showed considerable variation among clones for all the traits and this may be attributed to the larger number of clones included in the study.

In comparison, the symptomless trees were better than symptomatic trees in only three of the clones for BW. This agrees with the low percentage reduction of 2.5 for BW, indicating that CSSVD infection may have little influence on the trait. Variation among clones for BW showed that 92% of the symptomless trees and 82% of the symptomatic trees met the minimum BW value of 1.0 g established by the chocolate industry and could be sold on the international market. The lowest BW of clone SCA 6 for both symptomless (0.78 g) and symptomatic (0.72 g) trees corresponds to findings of [33] and [34] who reported BW of 0.71 g and 0.79 g for SCA 6, respectively and confirm previous reports [35–37] that BW is highly heritable.

For NoBP, the values obtained for majority of the clones were significantly higher in symptomless trees than symptomatic trees, indicating that CSSVD may influence the trait. [28, 38] concluded that the NoBP depends on a lot of factors including number of ovules per ovary, which is influenced by intensity of pollinating, and insects and disease infestation. The infected cacao trees lose chlorophyll as a result of vein-clearing and other symptoms which affect the plant’s photosynthetic efficiency [39], and subsequently reduce biomass and NoBP. With regards to DBY, variation among symptomless and symptomatic clones was generally high; a mean of 1.29 t/ha/year for symptomless and 1.01 t/ha/year for symptomatic with a mean difference of 0.28 t/ha/year. Dry bean yield is a component that depends on BW, NoBP and number of pods per tree. Although, the values of symptomless trees were higher than symptomatic trees for BW and NoBP, the magnitude in DBY was very large (0.28 t/ha/year). This may be accounted for by number of pods per tree which was always higher in symptomless trees compared to symptomatic trees.

This result is very consistent with those reported by [39] with regards to the difference between symptomless and symptomatic clones in experiments with Amelonado and Upper Amazon cacao clones, where a difference of 0.27 t/ha/year was observed between symptomless and symptomatic trees for Upper Amazon cacao clones and 0.41 t/ha/year for Amelonado cacao clones. The decrease in yield after infection, evident in 79% of the clones in this study, is a clear indication of the negative effects of the CSSVD on DBY. A decrease in yield in unhealthy cacao trees after infection with CSSVD was also observed by [40].

Out of the 20 best high yielding cacao clones identified (Tables 3 and 4) which recorded yields above 1.4 t/ha, eight (NA 124, T17/358, T35/78, T57/305, T63/971 x SCA 6, T65/239, T76/1835 and T82/2294) combined high yield with no DI. Clone T17/358 is an open pollinated clone of IMC 53, T63/971 is a hybrid of PA 35 x IMC 85, T65/239 is a hybrid of PA 7 x IMC 47, T76/1835 is a hybrid between PA 35 x NA 31 and T 82/2294 is a hybrid of NA 32 x PA 35. Even though they are all of Upper Amazon origin with the exception of T35/78 an unknown genotype and T57/305 a Trinitario, only T63/971 and T17/358 are used as parental clones in the Seed Gardens of Ghana for pod production. The remaining 12 clones (B 36, ICS 40, PA 150 x SCA 9, NA 33 x IMC 67, T29/378, T30/628, T39/651, T60/887, T63/971, T76/1136, T 81/1879 and T 82/503) among the 20 best high yielding clones even though are potential high yielding clones, some incidence of the CSSVD was observed on those clones during the study.

Based on the R^2^ value, the degree of covariation (coefficient of determination) between yield of symptomless trees and symptomatic trees is 62%. In this analysis, deviation of the R^2^ value from unity expresses the degree of yield losses due to CSSVD infection. Clones above the line in Fig 2 are less sensitive to disease infection in terms of yield than those below the line. The effect of CSSVD infection on clones could be grouped into two classes; high yielding clones with less yield reduction as a result of CSSVD infection (e.g. T60/887) and high yielding clones with high yield reduction as a result of CSSVD infection (e.g. T76/1136). For instance, clone T76/1136 invariably had 0.1 t/ha yields higher than T60/887, but the latter had yield reduction of only 0.16 t/ha whereas the former had 0.54 t/ha. These differences could enable the selection of high yielding varieties that are tolerant to CSSVD and eight of the high yielding clones; B 36, ICS 40, NA 33 x IMC 67, T30/628, T60/887, T63/971, T 81/1879 and T 82/503 had lower yield reductions. Clone T81/1879 is a hybrid of NA 32 x NA 31, T60/887 is a hybrid of PA7 x NA 32 and T 82/503 is a hybrid of NA 32 x PA 35. Among these, only T60/887 is used as parental clones in the Seed Gardens of Ghana for pod production, and the other seven identified above could also be used as parental clones in the seed garden.

Disease incidence was highly variable with some of the clones having all of their trees showing no CSSVD symptoms, others had small proportions of their trees showing CSSVD symptoms whereas others had large proportions showing CSSVD symptoms. Those with very high DI included CC 10, D 26, EET 399, EQX 3338, EQX 78, ICS 16, ICS 25, MOQ 210, P 16B, POUND 7 and T 60/1774 and could be concluded that these clones will be of little use in applied cacao breeding that seeks to develop tolerant CSSVD varieties. Clones that showed no CSSVD symptoms (CAM 12, O2, IMC 61, NA 124, NA 33, PA 118, PA124, PA 150 × SCA 6, T 9/22, T 12/151, T 17/358, T 20/126, T 20/50, T 35/78, T 57/305, T 60/887 × IMC 53, T 63/971 × SCA 6, T 65/239, T 65/326, T 76/1835, T 79/1150, T 82/2294, T 85/874, T 99/1614 and T 99/795) may possess CSSVD tolerance genes or may have escaped infection. It is worth noting that the majority of this group are from the Upper Amazon population. This concurs with previous studies [19, 41, 42] which concluded that among the different cacao genetic groups, the Upper Amazon clones are more tolerant to CSSVD infection.

Disease spread by the new Juabeng 1A strain virus (CSSV 1A) among the different genetic groups was lowest (26%) in Contanama clones and highest (44%) in Amelonado clones after 6 –year period. Although Contanama had only 5 clones from the onset of the study, this observation is consistent with regards to what was found by [41] who reported the lowest DI of 22% in SCA crosses and highest of 75% in Amelonado selfs over a four-year period of infection by the CSSV 1A. The differences in DI between both studies may partly be accounted by the differences in experimental designs, a single tree randomisation design in this study and block design in the previous study. Most of the spread of CSSVD in the field occurs in a radial manner as mealybugs move and feed in inter-locking branches of cacao canopy [11]. In a single tree randomisation design, trees of different clones are randomised within blocks and canopy of those that are resistant/tolerant within blocks may delay disease transmission by mealybugs from infected to healthy plants. In block planting, because plots are composed of the same genotypes, infected plants serve as sources of inoculum from which further spread by mealybugs occurred.

Disease incidence among the underrepresented groups of Guiana and Curaray was delayed until the second year before the trees started to express symptoms. Infections were also low, 16% in COCA 3348/52 and 14% in GU 219/V. These two clones could be used as parents to broaden the range of tolerant clones in breeding programmes that seek to develop CSSVD resistant varieties. Interestingly, Trinitario clones dominated both the low and high DI classes. This shows that in any CSSVD studies, the resistance levels of clones should be estimated and classified into susceptible or resistant before being applied in any breeding program. Trinitarios are natural hybrids between Forastaro and Criollo, and because Forasteros are Upper and Lower Amazon genotypes, such mixed reaction should be expected for clones of Trinitario origin. [40] reported high resistance when Trinitario clone T9/21 was used in crosses with Upper Amazon clones. However, [16] found low resistance when Trinitario clone ACU 85 was crossed with Upper Amazon clones.

Monitoring of CSSVD infection in this study was based on visual method of symptom expression and in cases where clones are latently infected, it is difficult to identify infected trees. A DNA-based PCR approach could have improved the reliability of the data in confirming the status of the symptomless trees. It is well established, however, that most of the CSSVD primers available are strain-specific and the detection power of these primers are very low, about 30% [43]. From the results of the index experiment, visual method used in monitoring disease infections in this study was high (92%). This implies that the visual method is quite reliable and could be used until reliable primers are made available.

## Conclusions

The significant differences between symptomless and symptomatic trees of the same clones identified, especially for yield suggests that yield component is important in selecting tolerant CSSVD materials. This implies that cacao clones should be tested extensively for those traits to select tolerant CSSVD clones that are high yielding to maximize yield. Cocoa swollen shoot virus disease studies for yield traits in cacao is very scarce and previous studies have used gauze-house screening method where several authors found that Upper Amazon clones are more tolerant than the Amelonaldo and Trinitario clones. In such studies, the Upper Amazon clones were recommended for planting to replace the Amalonado type which previously dominated (80–85%) cacao plantings in Ghana. Results from the study showed that there is considerable variation among the clones for DI and percentage yield reduction, and potential clones could be identified in the different genetic groups. Specific high yielding and CSSVD tolerant genotypes should therefore be the focus of selection. Clones COCA 3348/52 and GU 219/V among the underrepresented, B 36, ICS 40, NA 33 x IMC 67, T30/628, T60/887, T63/971, T 81/1879 and T 82/503 among those that combine high yield with low yield reduction, and NA 124, T17/358, T35/78, T57/305, T63/971 x SCA 6, T65/239, T76/1835 and T82/2294 among those that combine high yield with no-disease incidence are promising tolerant CSSVD cacao clones. Their addition to breeding programs that seek to develop resistant CSSVD varieties or being deployed as planting materials in endemic areas in Ghana and other West African cacao growing countries is recommended on the basis of the present observations. It is also recommended that large plantings should be composed of clones of different genetic backgrounds to delay or minimize disease spread.

## Supporting information

S1 Table Classification of 210 genotypes according to CSSVD incidence. (DOCX)

## References

[1] Aneani F, Anchirinah V, Asamoah M, Owusu-Ansah F. Baseline socio-economic and farm management’s survey. A Final Report for the Ghana Cacao Farmers’ Newspaper Project, 2007. Cacao Research Institute of Ghana (CRIG), New Tafo–Akim, Ghana. doi: 10.1094/PDIS-03-16-0404-RE

[2] Muller E. Cocoa swollen shoot virus. Chapter 24 in: Characterization, Diognosis and Management of plant viruses: Industrial crop 2008, Roa G. P., Paul Khurana S. M. and Lenardom S. L. eds. Studium Press, Houston, TX.

[3] Abrokwah F, Dzahini-Obiatey H, Galyuon I, Osae-Awuku F, Muller E. Geographical distribution of cacao swollen shoot virus molecular variability in Ghana. Plant Disease 2016; 100, 2011e2017. doi: 10.1094/PDIS-01-16-0081-RE 30682997

[4] Posnette AF. Virus diseases of cacao in West Africa I. Cacao viruses 1A, 1B, 1C and 1D. Annals of Applied Biology 1947; 37: 378–384. doi: 10.1111/j.1744-7348.1947.tb06372.x 20271954

[5] Posnette AF. The diagnosis of swollen shoot disease of cacao. Tropical Agriculture 1943; 21:156–158.

[6] Thresh JM. The availability of cacao swollen shoot virus to mealybug feeding on infected trees. Report of the West African Cocoa Research Institute 1957–58, pp 78–81.

[7] Thresh JM, Lister RM. Coppicing experiments on the spread and control of cacao swollen shoot virus disease in Nigeria. Annals of Applied Biology 1960; 48: 65–74.

[8] Amon-Armah F, Owusu D, Baah F, Owusu F. Farmers’ adoption of preventive and treatment measures of cocoa swollen shoot virus disease in Ghana. Journal of Agriculture and Food Research 2021; doi: 10.1016/j.jafr.2021.100112

[9] Dzahini-Obiatey H, Domfeh O, Amoah FM. Over seventy years of a viral disease of cocoa in Ghana: from research perspective. African Journal Agricultural Research 2010; 5: 476–485.

[10] Posnette AF. Virus research. Annual Report, West African Cocoa Research Institute 1948; 11: 1946–1947.

[11] Thresh JM, Owusu GK, Boamah A, Lockwood G. Ghanaian cocoa varieties and swollen shoot virus. Crop Protection 1988; 7: 219–231.

[12] Ollennu LA, Osei BK, Acheampong. The Use of Nonhost Crops as Barrier between Cocoa Plantings. Summary of Thrust Reports 1994–1995. p. 42. Cocoa Research Institute, Tafo, Ghana.

[13] Dzahini-Obiatey H, Ameyaw GA, Ollennu LA. Control of cocoa swollen shoot disease by eradicating infected trees in Ghana: a survey of treated and replanted areas. Crop Protection 2006; 25: 647–652.

[14] Domfeh O, Dzahini-Obiatey H, Ameyaw GA, Abaka-Ewusie K, Opoku G. Cocoa swollen shoot virus disease situation in Ghana: a review of current trends. African Journal Agricultural Research 2011; 6: 5033–5039.

[15] Owusu GK, Ollennu LA. The problems of the re-infection of replanted cocoa by cocoa swollen virus in Ghana. In: Proceedings of the First International Cocoa Pest and Diseases Seminar, Novotel, Accra, Ghana, November 6–10 1997, pp. 179–188.

[16] Padi FK, Domfeh O, Takrama J, Opoku SY. An evaluation of gains in breeding for resistance to the cocoa swollen shoot virus disease in Ghana. Crop Protection 2013; 51: 24–31.

[17] Crowdy SH, Ponette AF. Virus disease of cacao in West Africa. II Cross-immunity experiments with virus 1A, 1B and 1C. Annals of Applied Biology 1947; 34: 403–411. 10.1111/j.1744-7348.1947.tb06373.x 20271955

[18] Posnette AF, Todd JM. Virus diseases of cacao in West Africa. VIII. The search for virus-resistant cacao. Annals of Applied Biology 1951; 38: 785e800.

[19] Legg JT, Lockwood G. Evaluation and use of a screening method to aid selection of cocoa (*Theobroma cacao* L.) with field-resistance to cocoa swollenshoot virus in Ghana. Annals of Applied Biology 1977; 86: 241e248.

[20] Aikpokpodion PO, Motamayor JC, Adetimirin VO, Adu-Ampomah Y, Ingelbrecht I, Eskes A B, et al. Genetic diversity assessment of subsamples of cacao, *Theobroma cacao* L. collections in West Africa using simple sequence repeats marker. Tree Genetics and Genomics 2009; 5: 699–711. 10.1007/s11295-009-0221-1

[21] Opoku SY, Bhattacharjee R, Kolesnikova-Allen M, Motamayor JC, Schnell R, Ingelbrecht IL, et al. Assessment of genetic diversity and population structure in West African cacao: A case study on collections from Ghana. Journal of Crop Improvement, 2007; 20; 73–87. 10.1300/J411v20n01_04

[22] Baah F, Anchirinah V, Amon-Armah F. Soil fertility management practices of cacao farmers in the eastern region of Ghana. Agriculture and Biology Journal of North America 2011; 2: 173–181. 10.5251/abjna.2011.2.1.173.181.

[23] Padi FK, Ofori A, Auther A. Genetic variation and combining abilities for vigour and yield in a recurrent selection programme for cacao. Journal of Agricultural Science, 2016; doi: 10.1017/S00218596160004591–21

[24] Ameyaw GA, Dzahini-Obiatey H, Domfeh O. Perspectives on cocoa swollen shoot virus disease (CSSVD) management in Ghana. Crop Protection 2014; 65: 64–70.

[25] Andres C, Gattinger A, Dzahini-Obiatey H, Blaser WJ, Offei SK. Combatting cocoa swollen shoot virus disease: what do we know? Crop Protection 2017; 98: 76–84. 10.1016/j.cropro.2017. 03.010.

[26] Cheesman EE. Notes on the nomenclature, classification and possible relationships of cacao populations. Tropical Agriculture 1944; 21: 144–159.

[27] Motamayor JC, Lachenaud P, Mota SJW, Loor R, Kuhn DN, Brown S, et al, Geographic and genetic population differentiation of the Amazonian chocolate tree (*Theobroma cacao* L) *PLoS ONE* 2008; 3:10, 10.1371/journal.pone.0003311 PMC2551746 18827930

[28] Doaré F, Ribeyre F, Cilas C. Genetic and environmental links between traits of cocoa beans and pods clarify the phenotyping processes to be implemented. Scientific Reports 2020; 10: 9888, doi: 10.1038/s41598-020-66969-9 32555337 PMC7303165

[29] Lockwood G, Gyamfi MMO. The Cocoa Research Institute of Ghana cocoa germplasm collection with notes on codes used in the breeding programme at Tafo and elsewhere. Technical Bulletin No 10. 1979; New-Tafo, Akim, Ghana: Cocoa Research Institute of Ghana.

[30] Abdul-Karima A, Adomako B, Adu-Ampomah Y. Cocoa introduction into Ghana. Ghana Journal Agricultural Science 2006; 39:227–238.

[31] Glendinning DR. The performance of the introductions and hybrids in W.A.C.R.I. trials. In: Report Cocoa conference 1957, Cocoa, Chocolate and Confectionery Alliance, London, pp 41–44

[32] Ameyaw GA. Management of the Cacao Swollen Shoot Virus (CSSV) Menace in Ghana: The Past, Present and the future. 2019; 10.5772/intechopen.87009.

[33] Cilas C, Machado R, Motamayor JC. Relationship between several traits linked to sexual plant reproduction in *Theobroma cacao* L: Number of ovules per ovary, number of seed per pod, and seed weight. Tree Genetics and Genomes 2010; 6: 219–226. 10.1007/s11295-009-0242-9

[34] Ofori A, Padi FK, Ameyaw GA, Dadzie AM, Lowor ST. Genetic variation among cacao (*Theobroma cacao* L.) progenies for resistance to cacao swollen shoot virus disease in relation to total phenolic content. Plant Breeding 2015; 134: 477–484. doi: 10.1111/pbr.12282

[35] Lockwood G, Pang JTY. Cocoa Breeding at BAL Plantations: Genetic analysis and its implications for breeding strategy. In Proceedings of the International Workshop on Cocoa Breeding Strategies (INGENIC), 1995; 66–80. Kuala Lumpur, Malaysia, October 18–19, 1994.

[36] Oyedokun AV, Omoloye AA, Adewale BD, Adeigbe OO, Adenuga OO, Aikpokpodion PO. Phenotypic variability and diversity analysis of bean traits of some cocoa hybrids in Nigeria. Asian Journal of Agricultural Sciences, 2011; 3:127–31.

[37] Pardo J, Enriquez GA. Herencia de algunos components de la calidad industrial en almendras de cacao (*Theobroma cacao* L.). In Proceedings of the 10th International Cacao Research Conference, 1988; 695–99. Santo Domingo, Dominican Republic, May 17–23.

[38] Malebajoa AM, Karen JE, Francois R, Shayne MJ. Physiological responses to folivory and phytopathogens in a riparian tree, *Brabejum stellatifolium*, native to the fynbos biome of South Africa. African journal of Ecology 2018; 56:477–487.

[39] Brunt AA. The effects of cocoa swollen-shoot virus on the growth and yield of Amelonado and Amazon cocoa (*Theobroma cacao* L) in Ghana. Annals of Applied Biology 1975; 80: 169–180.

[40] Legg JT, Kenten RH. The resistance and tolerance of different cocoa varieties to cocoa swollen‐shoot virus in Ghana. Annals of Applied Biology 1970; 65: 425–434.

[41] Legg JT, Kenten RH. Field experiments on the resistance of cocoa to cocoa swollen‐shoot virus Annals of Applied Biology 1971; 67; 369–375.

[42] Legg JT, Lockwood G. Resistance of cocoa to swollen-shoot virus in Ghana. I. Field trials. Annals of Applied Biology 1981; 97: 75e89.

[43] Ameyaw GA, Chingandu N, Domfeh O, Dzahini-Obiatey HK, Gutierrez OA, Brown JK. Variable detection of Cacao swollen shoots disease-associated badnaviruses by PCR amplification. *Proceedings*, *International Symposium on Cocoa Research*, *T3-Pests and Diseases*. *Lima*, *Peru*, *13–17 Nov 2017*.

