# Supplementary material for: Field evaluation of the impact of cocoa swollen shoot virus disease infection on yield traits of different cocoa (Theobroma cacao L.) clones in Ghana
Source: PLoS One. 2022 Jan 20;17(1):e0262461. doi: 10.1371/journal.pone.0262461 (PMC8775274; doi:10.1371/journal.pone.0262461)
Supplement: S1 Table — (DOCX) [file pone.0262461.s001.docx]

**S1 Table. Classification of 210 genotypes according to CSSVD incidence.**

| No incidence (0%) | Low incidence (<25%) | Moderate incidence (25-50%) | Moderate incidence (25-50%) | High incidence (50-75%) | Very high incidence (>75%) |
| --- | --- | --- | --- | --- | --- |
| CAM 12 | AMAZON 3-2 | A 12 | T 3/247 | BE 10 | CC 10 |
| E 9 | C/SUL 7 | A 164/43 | T 30/539 | D 70 | D 26 |
| IMC 61 | EET 397 | A 45 | T 30/628 | E 75 | EET 399 |
| NA 124 | GU 123/C | A 46 | T 51/478 | GU147/C | EQX 3338 |
| NA 33 | ICS 70 | A 72 | T 53/46 | IMC 83 | EQX 78 |
| O2 | IMC 23 | B 36 | T 56/118 | K 5 | ICS 16 |
| PA 118 | IMC 60/112 | CC 11 | T 6/526 | MA 12 | ICS 25 |
| PA 150 × SCA 6 | K 9 | DOM 4 | T 60/887 | MAN 15-2 | MOQ 210 |
| PA124 | MO 20 | E 17 | T 60/887 × NA 33 | N 33/326 | P 16B |
| T 12/151 | NA 3 | GC 29 | T 61/1239 | N8/112 | POUND 7 |
| T 16/618 | NA 33 × PA 7 | GU125/C | T 63/882 | NA 227 | T 60/1774 |
| T 17/358 | NA 387 | GU219/V | T 63/971 | NA 33 x IMC 53 |  |
| T 20/126 | PA 107 | GU249/H | T 76/1068 | NA 33/326 |  |
| T 20/50 | PA 150 × IMC 67 | ICS 39 | T 79/1064 | NA 427 |  |
| T 24/297 | PA 16 | ICS 40 | T 79/380 | NA 904 |  |
| T 35/78 | PA 56 | ICS 43 | T 79/487 | PA 65 |  |
| T 49/778 | 15/5 | 18/11 | T 8/199 | PA 7 × IMC 67 |  |
| T 57/305 | PNG 10 | IMC 55 | T 8/287 | PASCAL |  |
| T 60/887 × IMC 53 | PNG 336 | IMC 6 | T 81/1879 | PENTAGONA |  |
| T 63/971 × SCA 6 | S 72 | IMC 67 | T 82/503 | SCA 6 |  |
| T 65/238 | SCA 9 | IMC 76 | T 85/799 × MA 12 | SGU 50 |  |
| T 65/239 | SPEC 160/9 | L6/428 | T 87/12 | T 13/382 |  |
| T 65/326 | T 11/94 | MO 9 | T 9/66 | T 16/530 |  |
| T 76/1835 | T 12/61 | MOQ6/95 | T 90/187 | T 39/651 |  |
| T 79/1150 | T 13/472 | NA 242 | T 90/2093 | T 4/159 |  |
| T 82/2294 | T 14/233 | NA 33 × IMC 67 | TF 6 | T 60/885 |  |
| T 85/874 | T 23/458 | NA 33 × T60/887 | Y 44 | T 63/967 |  |
| T 9/22 | T 24/229 | NA 585 | Z 47 | T 76/1224 |  |
| T 92/1614 | T 3/335 | NA 929 |  | U 7 |  |
| T 92/795 | T 43/1054 | P 30 |  |  |  |
|  | T 44/547 | PA 13 |  |  |  |
|  | T 44/600 | PA 150 |  |  |  |
|  | T 45/145 | PA 150 × NA 33 |  |  |  |
|  | T 57/308 | PA 150 × SCA 9 |  |  |  |
|  | T 6/525 | PA 150 × T60/887 |  |  |  |
|  | T 60/1052 | PA 37 |  |  |  |
|  | T 60/975 | PA 65 |  |  |  |
|  | T 61/1326 | PA 7 |  |  |  |
|  | T 62/205 | PA 7 × IMC 53 |  |  |  |
|  | T 63/762 | PA 7 × NA 33 |  |  |  |
|  | T 63/961 | PA 7 × PA 150 |  |  |  |
|  | T 63/971 × T 60/887 | PA 7 × T 60/887 |  |  |  |
|  | T 72/1768 | PA 70 |  |  |  |
|  | T 72/2388 | PD 12A |  |  |  |
|  | T 73/1931 | PNG 360 |  |  |  |
|  | T 73/2454 | RB 41 |  |  |  |
|  | T 81/1880 | RB 49 |  |  |  |
|  | T 85/799 | S 19 |  |  |  |
|  | T 87/368 | T 114/584 |  |  |  |
|  | T 87/98 | T 12/63 |  |  |  |
|  | T 89/158 | T 14/295 |  |  |  |
|  | T 90/118 | T 17/1856 |  |  |  |
|  | T 99/395 | T 23/509 |  |  |  |
|  | T 99/396 | T 26/277 |  |  |  |
|  | T57/ 368 | T 28/805 |  |  |  |
|  | TF 20 | T 29/378 |  |  |  |
